# Feasibility and preliminary efficacy of different intensities of functional training in elderly type 2 diabetes patients with cognitive impairment: a pilot randomised controlled trial

**DOI:** 10.1186/s12877-024-04698-8

**Published:** 2024-01-18

**Authors:** Majid Mardaniyan Ghahfarrokhi, Hossein Shirvani, Mostafa Rahimi, Behzad Bazgir, Alireza Shamsadini, Vahid Sobhani

**Affiliations:** 1 Department of Sport Science, Shahrekord University, Shahrekord, Iran; 2 Exercise Physiology Research Center, Life Style Institute, Baqiyatallah University of Medical Sciences, Tehran, Iran

**Keywords:** Functional training, Elderly, Type 2 diabetes, Cognition

## Abstract

**Background:**

Aging and type-2 diabetes (T2D) are the most important risk factors for cognitive impairment and Alzheimer’s disease. Exercise training is an effective, safe, and practical intervention in improving glucose metabolism, physical function, and cognitive disorders. This pilot study investigated the feasibility and preliminary efficacy of high-intensity low-volume (HIFT) vs. low-intensity high-volume (LIFT) functional training in elderly T2D patients with cognitive impairment.

**Methods:**

Forty-eight elderly T2D patients (31 female, 17 male, age 67.5 ± 5.8 years, MMSE score 18.8 ± 2.6, FBG 209.5 ± 37.9) were randomly assigned to HIFT, LIFT and control groups. Cognitive impairment was diagnosed with MMSE ≤ 23 based Iranian society. The SDMT, CVLT-II, BVMT-R, and Stroop tests were used to evaluated processing speed, learning, memory and attention respectively. Physical fitness tests include: tandem stance and walk test; TUG; 6MWT, 10MWT; SSST; 5TSTS; and hand grip was used to evaluated static and dynamic balance, agility, walking endurance, gait speed, lower limb function and lower and upper body strength respectively. As well as, Biochemical (FBG, insulin, HOMA-IR, HbA1c) and physiological outcomes (SBP, and DBP) were assessed. The HIFT group performed six weeks of functional training (three sessions per week) with 120–125% of the lactate threshold. The LIFT group performed six weeks of functional training (five sessions per week) with a 70–75% lactate threshold. Feasibility, safety, and acceptability of exercise programs were assessed at the end of the study.

**Result:**

HIFT showed a higher adherence rate (91% vs. 87.5%), safety, and acceptability compared to LIFT. MMSE and Stroop scores, 6MWT, FBG, insulin, HOMA-IR, HbA1c, SBP, and DBP significantly improved in HIFT (*all*, *P* ≤ 0.004) and LIFT (*all*, *P* ≤ 0.023). Changes in 6MWT, FBG, insulin, HOMA-IR, and HbA1c in HIFT (*all*, *P* ≤ 0.001) and LIFT (*all*, *P* ≤ 0.008) were significant compared to the control group. Changes in Stroop scores were significant only in the HIFT group compared to the control group (*P* = 0.013). SDMT, CVLT-II, BVMT-R, balance test, 10MWT, SSST, TUG and hang grip significantly improved only in HIFT (*all*, *P* ≤ 0.038).

**Conclusion:**

HIFT vs. LIFT is a safe, feasible, and effective approach for improving some aspects of physical, biochemical, and cognitive function in elderly T2D patients with cognitive impairment. This pilot study provides initial proof-of-concept data for the design and implementation of an appropriately powered randomised controlled trial (RCT) of HIFT vs. LIFT in a larger sample of elderly T2D patients with cognitive impairment.

**Trial registration:**

Randomized controlled trial (RCT) (Iranian Registry of Clinical Trials, trial registration number: IRCT20230502058055N1. Date of registration: 11/06/2023.

## Background

Aging, as one of the most sensitive periods of life, is associated with many unfortunate consequences, including the occurrence of metabolic and psychological diseases and movement disorders [1]. According to WHO statistics in 2015, the number of people aged ≥ 60 was approximately 900 million people, which will reach approximately 2 billion people by 2050 [2]. In Iran, according to the latest census in 2015, the population of elderly people aged ≥ 60 years was 9.28%, and it will be approximately 25 to 30% of the total population of the country in 2031 [3]. Aging is associated with an increased risk of metabolic diseases, including diabetes. The prevalence of type 2 diabetes (T2D) is directly related to increasing age [4]. The forecast from the WHO shows that in 2025, the population of elderly diabetics will reach 300 million people, and in some races, 50% of the elderly population will have diabetes [5]. One of the most important problems in the elderly are cognitive impairment, dementia, and finally Alzheimer’s disease. Several risk factors cause the occurrence and exacerbation of cognitive impairment in elderly individuals and one of the most important known risk factors is diabetes [6]. Recently, researchers have proposed that “type-3 diabetes” (T3D) is a neurological disease that represents the progression of T2D to Alzheimer’s disease [7]. T3D is a neurohormonal defect related to insulin signaling, which is associated with an 80% decrease in the number of insulin receptors in patients compared to healthy individuals [8]. To better understand this mechanism, the hypothesis of “metabolic cognitive syndrome” (MCS) can be proposed to justify the complex relationship between metabolic and cognitive disorders [9]. Physiological symptoms such as extracellular insoluble plaques, internal nerve nodules, loss of hippocampal neurons, a decrease in acetylcholine production and a decrease in glucose consumption in the cerebrum and hippocampus (along with memory loss) cause [10]. All these changes in the brain are the result of long-term dysregulation of insulin signaling and glucose metabolism [11]. Unlike to the usual medical interventions, which are associated with some side effects, it proved that a decrease in blood glucose and blood lipid profiles as a result of an improved lifestyle (physical activity and exercise along with a healthy diet), behavior patterns and body composition is cornerstone of diabetes management [12]. exercise training is an effective, safe, and practical intervention in the treatment of disorders caused by TD3, including glucose metabolism and insulin signaling [13], improving inflammation [14] and improving cognitive disorders [15].

Considering that the positive effects of aerobic [16], resistance [17], and combined [15] exercise training on cognitive and motor performance in elderly T2D patients with cognitive impairment have been investigated in a few limited studies, the need for more studies is increasingly apparent. To achieve reliable results in the field of the effect of exercise training in elderly T2D patients with cognitive impairment, a more precise exercise prescription is needed. It is recommended that for elderly individuals with T2D, exercise intensity is one of the most important exercise variables that must be carefully controlled [18, 19]. Some studies show that high-intensity exercises have more effects on the cognitive performance of elderly individuals with diabetes [15, 16]. On the other hand, it has been reported in a review study that for elderly individuals with cognitive disorders, an exercise program with shorter sessions and more sessions is more suitable [20]. As well as, it shown that eight sessions of dynamic sitting exercises improves cognitive performance and quality of sleep in older adults with cognitive impairment [21]. Although, both aerobic and resistance exercises improve some aspects of cognitive and motor performance [22]. However, combined exercises (aerobic and resistance) did not improve all cognitive and motor functions [15]. But, it seems that functional exercises can improve the cognitive and motor performance of the elderly due to the creation of more mental challenges. Functional training is a special rehabilitation intervention that is used in more realistic environments to improve the performance of daily activities and includes aerobic exercises, resistance exercises, balance exercises, proprioceptive integration exercises, body positioning exercises, and core muscle stability exercises [23, 24]. A growing body of evidence shows how functional exercises create adaptation in the structure and function of the brain [25, 26]. In general, exercise training is very important for elderly T2D patients to improve their cognitive and functional status. However, the exercise program for these people should be carefully designed and recommended in terms of intensity, duration, type, and length of the course. Therefore, the aim of this pilot study is to investigate the feasibility and preliminary effectiveness of six weeks of different intensities of functional exercises in elderly T2D patients with cognitive impairment.

## Method

### Design

The Ethics Committee of Baqiyatallah University of Medical Sciences, Tehran, Iran (IR.BMSU.BAQ.REC.1401.109) and Iranian Registry of Clinical Trials (IRCT20230502058055N1; Date of registration: 11/06/2023) approved this pilot study involved feasibility and preliminary efficacy and performed based on the Declaration of Helsinki. The subjects all signed a written letter of consent.

### Participants

Elderly patients with T2D were recruited from the Shahrekord Diabetes Association, Shahrekord, Iran. The inclusion criteria included elderly (male/females) age ≥ 60 years, cognitive impairment/dementia (Mini–Mental State Examination (MMSE) score > 23, fasting blood glucose (FBG) ≥ 126 mg/dL (7.0 mmol/L)), diagnosis of diabetes (hemoglobin A1c (HbA1c) ≥ 6.5% (48 mmol/mol), based on the Iranian population [27]) and ability to walk without assistance. The exclusion criteria included diabetic foot ulcer, smoking and alcohol consumption, cancer, participation in regular exercise programs or drug interventions (such as anti-Alzheimer’s drugs), and surgery. Finally, the sample size of 48 (16 per group) was selected following other pilot studies of exercise intervention in the elderly population [28, 29] and pilot feasibility trials [30, 31].

### Randomisation

**Fig. 1:**
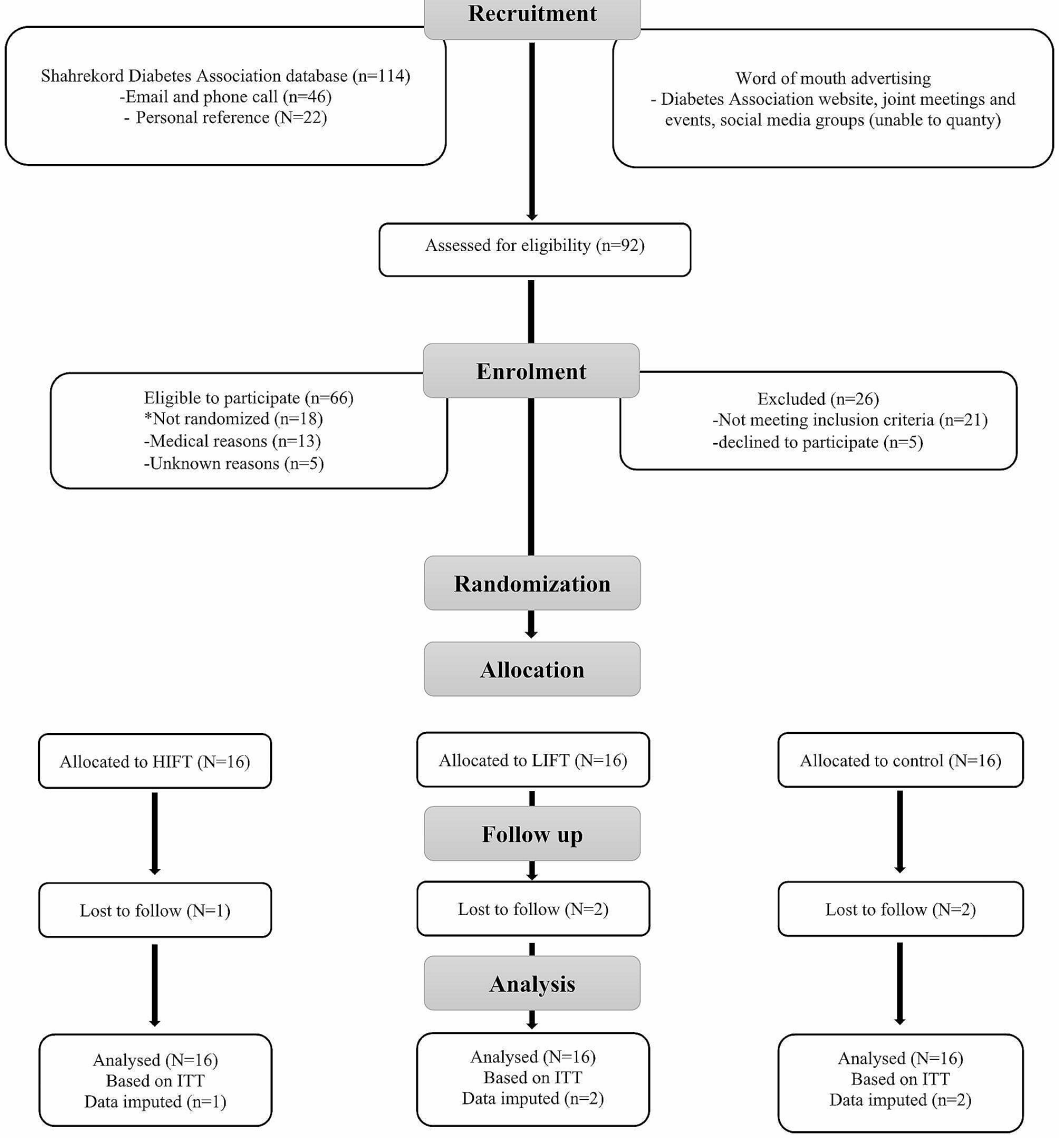
Study flowchart

To generate the randomisation scheme, the website Randomisation.com was used (http://www.randomisation.com). Subjects were randomly assigned into high-intensity functional training (HIFT), low-intensity functional training (LIFT) and control groups with randomisation stratified by MMSE scores, age, and blood glucose. Subjects were randomised to groups based on a block randomisation method. The subjects were allocated to a 2:2:2 ratio into conditions (Fig. 1). The MMSE score and other clinical measurements were determined using an expert physiologist with no role in random allocation. Following the primary examinations, the subjects were assigned with equal probability to the HIFT, LIFT, and control groups. The randomisation allocation table was generated by a member of the team blinded to the assessments. Afterward, the allocation numbers were placed envelopes and sealed.

The age and duration of the disease were extracted from the patients’ medical records. Height was measured by a portable height measuring meter wall mounted retractable, and weight, fat mass, body mass index (BMI), and lean mass were measured using an InBody-570 made in Korea.

### Modified Bruce test

Maximum oxygen consumption and maximum heart rate were evaluated using the modified Bruce test (recommended for the elderly) [32]. To examine the maximum oxygen consumption and maximum heart rate, the modified Bruce protocol was employed. Compared to the standard Bruce test, the modified version starts at a lower workload and it is usually utilized for sedentary and old age patients. The starting two stages of the test are carried out at 1.7mph and 0% grade and 1.7mph and 5% grade. The third stage is similar to the first stage of the standard version as mentioned above. VO_2max_ was measured by following the formula based on the result of the modified Bruce protocol. Additionally, the subject’s maximum heart rate during exhaustion was measured [32].

$$\eqalign{& {\rm{Women: V}}{{\rm{O}}_{{\rm{2max}}}}{\rm{}}\left( {{\rm{ml/kg/min}}} \right)\\&= 4.38 \times {\rm{ T }} - 3.9\;\;\;{\rm{Men: V}}{{\rm{O}}_{{\rm{2max}}}}\left( {{\rm{ml/kg/min}}} \right)\\&= 2.94 \times {\rm T }+ 7.65}$$

### Cognition and dementia

The MMSE test was used to diagnose and screen cognitive impairment and dementia. The MMSE is a test to evaluate the quality of consciousness by diagnosing and screening dementia, the maximum score of which is 30. Based on the scores obtained for Iranian society, a score of less than 23 on the MMSE test indicates dementia or cognitive impairment [33]. Through the Symbol Digit Modalities Test (SDMT) processing speed was evaluated [34], using the California Verbal Learning Test Second Edition (CVLT-II) learning was examined [35], utilizing the Brief Visuospatial Memory Test-Revised (BVMT-R) memory evaluated [36], and using the Stroop Color and Word Test (SCWT) attention was examined [37].

### Functional tests

Static and dynamic balance was measured by a tandem stance group test that was examined using 10 positions. The subjects were tested after standing still for 20 s [38]. Test positions included (1) Semitandem stance, (2) Tandem stance, (3) Wide stance, (4) Narrow stance, 5,6) Right and/or left leg stance with eyes closed, 7) Tandem walk (dynamic balance); walk with feet with tandem position that is with the heel of one foot in front of and touching the toes of the other foot around the 3 m straight line, 8,9) Right and/or left leg stance with eyes open, 10) Feet tandem with eyes closed. It is notable, that it would be considered an error when if the person steps out of the position or a step in which the heel of the front does not touch the toes of the other foot and for each error one second would be added.

To assess gait speed, the ten-meter walking test (10MWT), walking endurance, six-minute walking test (6MWT) [39], and lower extremity (risk of falling) six spot step test (SSST) [40] were used. The timed up-and-go test (TUG) was employed to examine reaction time [39]. Hand grip [41] and the five-time sit-to-stand test (5TSTS) [42] were used to assess upper and lower body strength.

### Biochemical and physiological measures

Twenty-four hours prior to the exercise protocol and 48 h following the final session blood samples (10 mL) from the antecubital vein were collected in a sitting position with a 12-hour fasting state. Measuring FBG was carried out using a glucose oxidase method kit (Pars Azmoon, Iran) using autoanalyzer devices (Hitachi^®^, 704, 902, Japan). To measure serum insulin concentration, enzyme-linked immunosorbent assay (ELISA) was used with a microplate reader. To determine homeostasis model assessment of insulin resistance (HOMA-IR), the following formula was used: (fasting glycemia [mmol/L]×fasting insulin [mIU/L])/22.5 [43]. Those who had used insulin injection did not undergo the HOMA-IR analysis. HbA1c was measured by immunoturbidometry. Systolic (SBP) and diastolic blood pressure (DBP) were determined using a digital sphygmomanometer after 15 min of rest in stable conditions.

All measures were assessed 24 h before treatment (baseline) and 48 h following the intervention (follow-up). A research assistant blinded to the grouping, carried out all the baseline and following measurements.

### Exercise training interventions

Subjects in the HIFT group performed functional training with an intensity higher than the lactate threshold (75–85% of the heart rate reserve (HRR), equivalent to 80–85% of the maximum oxygen consumption, equivalent to 120–125% of the lactate threshold) [44–46]. Subjects were first to undergo a one-week acquaintance training course (three sessions per week) under direct supervision to learn about functional exercises and the gym environment and familiarise themselves with the equipment. Then, for six weeks (three sessions per week), they continued functional exercises while being supervised by physical trainers. Each HIFT session consists of 30–35 min of activity, which will include 1- endurance exercises, 2- upper and lower body strength, 3- balance exercises and maintaining posture, and 4- hip control exercises and mid-body stability. The ratio of rest to activity time in this group was 1:1. (Table 1).

Subjects in the LIFT group performed functional training with an intensity lower than the lactate threshold (35–45% of HRR, equivalent to 50–60% of maximal oxygen consumption, equivalent to 70–75% of lactate threshold) [44–46]. Subjects first undergo a one-week acquaintance training course (five sessions per week) under direct supervision to learn about functional exercises and the gym environment and familiarise themselves with the equipment. Then, for six weeks (five sessions each week), they continued functional exercises while being supervised by a physical trainers. The ratio of rest to activity time in the LIFT group was 3:1. Therefore, the approximate time of each session was between 40 and 45 min.

Ten minutes of warm-up before each session and five minutes of cool-down at the end of each session were considered. The heart rate was monitored by a polar heart rate monitor to control the training intensity during the training sessions. Additionally, lactate levels were evaluated after the end of each session to determine and adjust the level of intensity of exercise training (Table 1).

The subjects in the control group had no exercise intervention while in the study.

**Table 1:** Exercise protocol

	Sessions (HIFT)	Sessions (LIFT)	Functional training
Week 1–2	• S1-3 (25–30 min per session) • 10–12 Rep • 75% HRR • 1:1 rest to active ratio	• S1-5 (35–40 min per session) • 7–9 Rep • 35% HRR • 1:3 rest to active ratio	$\checkmark$ Endurance training $\checkmark$ Posture and Balance exercises $\checkmark$ upper and lower-extremity strength $\checkmark$ Pelvic control exercises and core stability training
Week 3–4	• S1-3 (25–30 min per session) • 12–14 Rep • 80% HRmax • 1:1 rest to active ratio	• S1-5 (35–40 min per session) • 8–10 Rep • 40% HRR • 1:3 rest to active ratio	$\checkmark$ Endurance training $\checkmark$ Posture and Balance exercises $\checkmark$ upper and lower-extremity strength $\checkmark$ Pelvic control exercises and core stability training
Week 5–6	• S1-3 (30–35 min per session) • 10–12 Rep • 85% HRR • 1:1 rest to active ratio	• S1-5 (40–45 min per session) • 7–9 Rep • 45% HRR • 1:3 rest to active ratio	$\checkmark$ Endurance training $\checkmark$ Posture and Balance exercises $\checkmark$ upper and lower-extremity strength $\checkmark$ Pelvic control exercises and core stability training

### Feasibility metrics

After six weeks, the subjects were asked to fill out a questionnaire with 12 items about acceptability and opinions about treatment. The data collected through this provided an insight about the intervention. The feasibility of the interventions was examined in the four domains such as retention and recruitment, resources such as costs and communication, scientific results such as burden, safety, experience, adherence, and the effects of treatment, and management such as safety reporting and data management. The results are listed in Table 2.

**Table 2:** Measures of feasibility for assessment strategy, monitoring and methodology of study protocol

Metrics	Monitoring and strategy	Method of assessment
Process -Recruitment and retention	-Recruitment and retention rate - Retention & attrition	The participants were recruited using telephone calls, email, word of mouth promotion, and brochures. The potential participants were entered into Excel software. In addition, the subjects recruitment flow and program section were input in Excel software.
Resources -Monetary and communication needs of the study	- Communication with participants - Costs of research	All connections with participants were added to Microsoft Excel. There were not any connection challenges during the six weeks of treatment. - All the expenses of the study was calculated.
Management - Safety report and data management of the study.	-SKU approval procedures - Preparing the staff and report time for participant communication - accuracy and time in data collection/entry	- All communications between staff and BMSU and time gap between MBSU submission and approval were - All call time, preparation, and time of taking reports during the program were input in Excel. - Additional data such as record time to collect, completeness, enter, and check data was added into Microsoft Excel.
Scientific -Safety, burden, adherence, experience, and treatment effect	- Serious adverse events (SAE)handling and reporting adverse events (AE), and clinical emergencies - Clinical emergencies, AEs, and SAEs. - Adherence, participants’ burden, and experience in the program - Treatment effect	- The subjects were required to record and report medical issues to the coaches. In addition there were required to fill out a questionnaire about their history of exercise treatments. The provided answers were recorded. -The subject reported their adverse events, exercise sessions, and experiences in log books for further discussion during the visits. - The clinical meaningfulness and effect size of any change in functional, biochemical, and cognitive outcomes were determined.

### Statistical analysis

Descriptive analyses including mean score and SD were reported for baseline and follow-up. Baseline specifications were compared using one-way ANOVA to determine similarity between the groups. In addition, the Shapiro‒Wilk test and frequency distributions were carried out to examine variance to fulfil the necessary assumptions for performing parametric statistical analysis for continuous variables. A two-way mixed-factor ANOVA (2 times×3 groups) was used to find the main differences following six weeks of intervention. To determine significant interaction effect, Bonferroni’s method was applied. Cohen’s *d* effect size (*d*) was also used to measure effect size (ES) in t tests. An intention-to-treat (ITT) analysis was performed, so that for missing data (lost to follow-up), multiple imputations were carried out. Data analysis was done in SPSS (v.24) (*p* < 0.05).

### Result

We assessed 63 diabetic elderly individuals for eligibility in a diabetes clinic (the Shahrekord Diabetes Association, Shahrekord, Iran). Following the preliminary visit, 48 subjects (31 female, 17 male, age 67.5 ± 5.8 years, MMSE score 18.8 ± 2.6, FBG 209.5 ± 37.9) were recruited. The subjects were randomly allocated to groups: 16 were allocated to HIFT, 16 to LIFT and 16 to control. No significant differences were observed in disease-modifying therapies or symptomatic therapies between the group before the intervention nor was there any changes in pharmacological treatments in the study. Five subjects (one subject from HIFT, two subjects from LIFT, and control) were excluded during the 6 weeks of training. The clinical study flowchart is illustrated in Fig. 1.

Table 3 lists the clinical and demographic specifications of the subjects. In short, the mean age of the subjects is 67.8 (SD = 5.7) years and the majority were women (65% of the sample). All subjects had T2D (100%) and suffered from impairment (MMSE: 19.4 (3.3)). No significant differences was observed between the subjects before the intervention in terms of age (*P* = 0.784), height (*P* = 0.413), weight (*P* = 0.388), BMI (*P* = 0.442), FBG (*P* = 0.559), insulin (*P* = 0.885), HbA1c (*P* = 0.731), SBP (*P* = 0.506), DBP (*P* = 0.572) or MMSE score (0.724) between the study groups.

**Table 3:** Baseline demographic and clinical characteristics of subjects in the study group

Variable	HIFT	LIFT	Control	*P* value
Age	66.47 ± 6.61	68.35 ± 5.44	67.76 ± 5.49	0.784
Height	164.33 ± 5.42	167.77 ± 6.38	165.21 ± 6.16	0.413
Weight	76.13 ± 7.76	74.35 ± 4.19	78.67 ± 5.54	0.388
BMI	28.25 ± 1.85	26.36 ± 2.31	28.86 ± 2.04	0.442
FBG (mg/dl)	208.75 ± 38.42	214.33 ± 35.24	205.58 ± 40.11	0.559
Insulin (mu/l)	10.10 ± 1.69	10.82 ± 1.46	9.96 ± 1.74	0.885
HOMA-IR	5.21 ± 1.33	5.73 ± 1.41	4.97 ± 1.38	0.611
HbA1c %	8.88 ± 1.91	9.07 ± 2.03	8.85 ± 1.85	0.731
SBP	146.14 ± 15.88	141.76 ± 16.13	142.24 ± 11.33	0.506
DBP	94.65 ± 5.33	92.54 ± 6.11	93.34 ± 6.08	0.572
MMSE	18.62 ± 2.84	19.08 ± 2.45	18.86 ± 2.57	0.724

HIFT: high-intensity functional training; LIFT: low-intensity functional training; BMI: body mass index; FBG: fasting blood glucose; HOMA-IR: homeostasis model assessment of insulin resistance; HbA1c: hemoglobin A1c; SBP: systolic blood pressure; DBP: diastolic blood pressure; MMSE: Mini–Mental State Examination

### Scientific feasibility: burden and experience

The subjects in HIFT (*n* = 16) and LIFT (*n* = 16) filled out the questionnaire. Based on VAS (visual analogue scale 0–10), the subjects in HIFT indicated a positive global rating on treatment received (9.5) vs. LIFT (8.8). They also stated that the duration of the study was enough (9.1 vs. 8.4), and found the objective feasible (9.2 vs. 7.4). In addition, they noted that the feedback given during the intervention was clear (9.4 for both treatment) and that they found the training enjoyable (9.2 vs. 8.3) and felt satisfaction with the outcomes (9.6 vs. 8.7). They reported tiredness after each session (8.1 vs. 7.4), and highlighted the necessity for more rests (2.8 vs. 2.9). The subjects in HIFT group mentioned that lower body practices (80.2%) with the highest yield, and core practices (28.5%) with the lowest yield. In comparison, subjects in LIFT group mentioned that the core body practices (62%) had the best outcomes and lower body practices (33.5%) had the poorest results.

### Adherence rate

The rate of participation in the HIFT group was 91% (262 of 288 sessions) for six weeks; this figure for the LIFT group was 87.5% (420 of 480 sessions).

### Safety

No adverse (serious or otherwise) events were reported by the LIFT groups; while HIFT participation reported some mild pain, muscle cramps, and fatigue. These reports were directly examined (via researcher) and indirectly checked (patients were asked to contact staff).

### Satisfaction with study components

All the subjects (*n* = 32, 100%; *n* = 16 of HIFT and *n* = 16 of LIFT) mentioned that the study coordinator, training sessions, and exercise manual were essential for the participations. In addition, they stated their satisfaction with the study coordinator, training sessions, and the intervention overall.

### Cognition outcomes

Two-way mixed-factor analysis showed a significant time main effect (*p* = 0.035) but not a group main effect (*p* = 0.306) or group by time interaction effect (*p* = 0.122) for the MMSE score. When examining within-group changes, we observed a significant increase in MMSE scores for the HIFT (*ES* = 0.73, *95% CI*, 0.57, 0.88, *P* = 0.004) and LIFT groups (*ES* = 0.71, *95% CI*, 0.55, 0.87, *P* = 0.007) but not for the control group (*ES=* -0.09, *95% CI*, -0.24, 0.07, *P* = 0.781). Effect size estimates for the MMSE score demonstrated that the overall effect size and the lower and upper confidence interval bounds for the HIFT and LIFT groups were “*moderate*,” while the nonsignificant changes for the control group were deemed “*trivial*”.

There were significant time main effects (*p* = 0.044) and group by time interaction effects (*p* = 0.032) but not group main effects (*p* = 0.077) for the Stroop test. Within-group analyses demonstrated a significant increase in Stroop scores for the HIFT (*ES* = 0.82, *95% CI*, 0.68, 0.98, *P* = 0.001) and LIFT groups (*ES* = 0.72, *95% CI*, 0.56, 0.88, *P* = 0.005) but not for the control group (*ES=* -0.24, *95% CI*, -0.44, -0.13, *P* = 0.236). Effect size estimates for the Stroop score demonstrated that the overall effect size and the lower and upper confidence interval bounds for HIFT were “*large*,” and those for the LIFT group were “*moderate*,” while the nonsignificant changes for the control group were deemed “*small*.” Between-group comparisons demonstrated that Stroop score changes in the HIFT group were significant vs. the control group (*P* = 0.013).

There were no significant group and time main effects or group by time interaction effects on SDMT, CVLT-II, or BVMT-R scores (*p* > 0.05). Within-group analyses demonstrated a significant increase in the SDMT score for HIFT (*ES* = 0.55, *95% CI*, 0.40, 0.71, *P* = 0.032) but not for LIFT (*ES* = 0.22, *95% CI*, -0.07, 0.38, *P* = 0.287) or control (*ES* = 0.08, *95% CI*, -0.07, 0.24, *P* = 0.794). Within-group analyses demonstrated a significant increase in the CVLT-II score for HIFT (*ES* = 0.61, *95% CI*, 0.42, 0.80, *P* = 0.019) but not for LIFT (*ES* = 0.42, *95% CI*, 0.26, 0.57, *P* = 0.141) or control (*ES* = 0.06, *95% CI*, -0.12, 0.25, *P* = 0.887). Within-group analyses demonstrated a significant increase in BVMT-R scores for HIFT (*ES* = 0.66, *95% CI*, 0.51, 0.82, *P* = 0.012) but not for LIFT (*ES* = 0.45, *95% CI*, 0.2296, 0.60, *P* = 0.127) or control (*ES=* -0.15, *95% CI*, -0.31, 0.00, *P* = 0.476). Effect size estimates for SDMT, CVLT-II, and BVMT-R scores demonstrated that the overall effect size and the lower and upper confidence interval bounds for HIFT were “*moderate*,” while the nonsignificant changes for the LIFT group were “*small*,” and the control group were deemed “*trivial*.” (Table 4).

### Fitness outcomes

There was only a significant time main effect (*p* = 0.001), group main effect (*p* = 0.006), or group by time interaction effect (*p* = 0.001) for the 6MWT. When examining within-group pre-post changes, we observed a significant increase in the 6MWT for the HIFT (*ES* = 0.91, *95% CI*, 0.75, 1.07, *P* > 0.001) and LIFT groups (*ES* = 0.85, *95% CI*, 0.69, 1.01, *P* > 0.001) but not for the control group (*ES=* -0.11, *95% CI*, -0.26, 0.04, *P* = 0.502). Effect size estimates for the 6MWT demonstrated that the overall effect size and the lower and upper confidence interval bounds for the HIFT and LIFT groups were “*large*,” while the nonsignificant changes for the control group were deemed “*trivial*.” Between-group comparisons demonstrated that 6MWT changes in the HIFT and LIFT groups were significant vs. the control group (*both*, *P* = 0.001).

There were no significant group and time main effects or group by time interaction effects on other fitness outcomes (*p* > 0.05). On the other hand, within-group analyses demonstrated a significant improvement in static balance for HIFT (*ES* = 0.50, *95% CI*, 0.34, 0.65, *P* = 0.47) but not for LIFT (*ES* = 0.32, *95% CI*, 0.16, 0.47, *P* = 0.404) or control (*ES* = 0.01, *95% CI*, -0.15, 0.16, *P* = 0.901). Dynamic balance showed a significant improvement for HIFT (*ES=* -0.58, *95% CI*, -0.73, -0.42, *P* = 0.023) but not for LIFT (*ES=* -0.25, *95% CI*, -0.40, -0.09, *P* = 0.218) or control (*ES* = 0.02, *95% CI*, -0.13, 0.18, *P* = 0.868). The 10MWT significantly improved the HIFT (*ES=* -0.53, *95% CI*, -0.58, -0.47, *P* = 0.035) but not the LIFT (*ES=* -0.21, *95% CI*, -0.36, -0.05, *P* = 0.254) or control (*ES=* -0.07, *95% CI*, -0.22, 0.09, *P* = 0.823). The SSST-dominant group demonstrated significant improvement in HIFT (*ES=* -0.58, *95% CI*, -0.73, -0.42, *P* = 0.025) but not in LIFT (*ES=* -0.29, *95% CI*, -0.45, -0.17, *P* = 0.184) or control (*ES=* -0.02, *95% CI*, -0.17, 0.14, *P* = 0.877). TUG significantly improved HIFT (*ES=* -0.54, *95% CI*, -0.59, -0.38, *P* = 0.029) but not LIFT (*ES=* -0.41, *95% CI*, -0.56, -0.25, *P* = 0.133) or control (*ES* = 0.09, *95% CI*, -0.06, 0.24, *P* = 0.565). Hand grip-dominant showed a significant improvement in HIFT (*ES* = 0.54, *95% CI*, 0.39, 0.70, *P* = 0.031) but not in LIFT (*ES* = 0.39, *95% CI*, 0.24, 0.55, *P* = 0.159) or control (*ES* = 0.02, *95% CI*, -0.13, 0.18, *P* = 0.885). The hand-grip-nondominant showed a significant improvement in HIFT (*ES* = 0.52, *95% CI*, 0.36, 0.68, *P* = 0.038) but not in LIFT (*ES* = 0.50, *95% CI*, 0.34, 0.66, *P* = 0.053) or control (*ES=* -0.12, *95% CI*, -0.28, 0.03, *P* = 0.492). Effect size estimates for static and dynamic balance, 10MWT, SSST-dominant, TUG, and hand grip (both domains) demonstrated that the overall effect size and the lower and upper confidence interval bounds for HIFT were “*moderate*,” while the nonsignificant changes for the LIFT group were “*small*,” and the control group was deemed “*trivial*.” There were no within-group pre-post changes for SSST-dominant and 5TSTS. (Table 4).

**Table 4:** Pre-post changes in cognitive and fitness outcomes in the study groups

	Pre-test Mean(SD)	Post-test Mean(SD)	d effect size (95% CI)	*P*-value	F
**Cognition outcomes** ***MMSE score***					
HIFT	18.62 ± 2.84	20.45 ± 2.16	0.73 (0.57 to 0.88)†	Group = 0.306 **Time = 0.035 #** Interaction = 0.122	0.807 **4.762** 1.314
LIFT	19.08 ± 2.45	20.83 ± 2.47	0.71 (0.55 to 0.87) †		
Control	18.86 ± 2.57	18.63 ± 2.81	-0.09 (-0.24 to 0.07)		
***SDMT***					
HIFT	27.35 ± 9.45	32.40 ± 8.79	0.55 (0.40 to 0.71) †	Group = 0.754 Time = 0.115 Interaction = 0.221	0.102 2.337 1.572
LIFT	25.94 ± 11.08	28.33 ± 10.18	0.22 (-0.07 to 0.38)		
Control	28.26 ± 9.94	29.04 ± 9.31	0.08 (-0.07 to 0.24)		
***CVLT-II***					
HIFT	8.42 ± 1.89	9.59 ± 1.84	0.61 (0.42 to 0.80) †	Group = 0.745 Time = 0.345 Interaction = 0.445	0.115 0.704 0.607
LIFT	9.13 ± 1.72	9.88 ± 1.89	0.42 (0.26 to 0.57)		
Control	8.86 ± 1.96	8.98 ± 1.85	0.06 (-0.12 to 0.25)		
***BVMT-R***					
HIFT	6.33 ± 1.75	7.55 ± 1.92	0.66 (0.51 to 0.82) †	Group = 0.538 Time = 0.452 Interaction = 0.309	0.355 0.597 1.008
LIFT	6.54 ± 1.83	7.34 ± 1.76	0.45 (0.29 to 0.60)		
Control	7.02 ± 1.67	6.82 ± 0.84	-0.15 (-0.31 to 0.00)		
***Stroop (Number correct)***					
HIFT	46.58 ± 10.35	54.67 ± 9.46	0.82 (0.66 to 0.98) † *	Group = 0.077 **Time = 0.044 #** **Interaction = 0.032 #**	1.479 **3.871** **4.908**
LIFT	49.33 ± 8.76	55.19 ± 7.44	0.72 (0.56 to 0.88) †		
Control	48.72 ± 11.55	45.27 ± 12.31	-0.29 (-0.44 to -0.13)		
**Fitness outcomes** ***Static balance (s)***					
HIFT	11.55 ± 5.68	14.18 ± 4.87	0.50 (0.34 to 0.65) †	Group = 0.565 Time = 0.230 Interaction = 0.620	0.445 1.559 0.199
LIFT	12.41 ± 4.21	13.88 ± 5.04	0.32 (0.16 to 0.47)		
Control	11.08 ± 6.24	11.12 ± 6.54	0.01 (-0.15 to 0.16)		
***Dynamic balance (s)***					
HIFT	24.39 ± 8.08	20.10 ± 6.71	-0.58 (-0.73 to -0.42) †	Group = 0.218 Time = 0.155 Interaction = 0.443	1.504 1.845 0.659
LIFT	22.16 ± 9.31	20.02 ± 7.98	-0.25 (-0.40 to -0.09)		
Control	23.41 ± 11.19	23.65 ± 9.74	0.02 (-0.13 to 0.18)		
***10MWT (s)***					
HIFT	21.37 ± 6.32	18.21 ± 5.66	-0.53 (-0.58 to -0.47) †	Group = 0.109 Time = 0.381 Interaction = 0.129	2.197 0.623 1.959
LIFT	20.13 ± 5.11	19.08 ± 4.83	-0.21 (-0.36 to -0.05)		
Control	23.28 ± 5.34	22.90 ± 6.13	-0.07 (-0.22 to 0.09)		
***6MWT (m)***					
HIFT	355.42 ± 67.54	422.10 ± 55.68	0.91 (0.75 to 1.07) †*	**Group = 0.006 #** **Time = 0.001 #** **Interaction = 0.001#**	**9.221** **13.205** **15.718**
LIFT	342.22 ± 87.49	408.75 ± 68.27	0.85 (0.69 to 1.01) †*		
Control	361.78 ± 58.51	355.42 ± 61.19	-0.11 (-0.26 to 0.05)		
***SSST dominant (s)***					
HIFT	18.86 ± 7.21	16.23 ± 6.05	-0.39 (-0.55 to -0.24)	Group = 0.866 Time = 0.451 Interaction = 0.732	0.021 0.599 0.089
LIFT	18.42 ± 6.28	17.04 ± 6.12	-0.22 (-0.38 to -0.07)		
Control	17.65 ± 5.86	17.88 ± 4.92	0.04 (-0.11 to 0.20)		
***SSST non-dominant (s)***					
HIFT	20.13 ± 4.54	17.61 ± 5.03	-0.58 (-0.73 to -0.42) †	Group = 0.202 Time = 0.298 Interaction = 0.126	1.559 1.228 1.917
LIFT	19.61 ± 5.78	18.05 ± 4.83	-0.29 (-0.45 to -0.14)		
Control	21.95 ± 4.98	21.87 ± 5.42	-0.02 (-0.17 to 0.14)		
***TUG (s)***					
HIFT	12.77 ± 4.65	10.24 ± 4.79	-0.54 (-0.69 to -0.38) †	Group = 0.877 Time = 0.188 Interaction = 0.122	0.019 1.762 2.314
LIFT	12.24 ± 5.04	10.29 ± 4.56	-0.41 (-0.56 to -0.25)		
Control	11.06 ± 4.37	11.48 ± 5.01	0.09 (-0.06 to 0.24)		
***Hand grip Dominant (kg)***					
HIFT	23.33 ± 8.76	27.51 ± 6.47	0.54 (0.39 to 0.70) †	Group = 0.639 Time = 0.201 Interaction = 0.344	0.385 1.617 0.802
LIFT	24.08 ± 7.42	27.12 ± 8.05	0.39 (0.24 to 0.55)		
Control	24.16 ± 6.45	24.33 ± 7.14	0.02 (-0.13 to 0.18)		
***Hand grip non-dominant (kg)***					
HIFT	22.77 ± 6.45	25.81 ± 5.18	0.52 (0.36 to 0.68) †	Group = 0.711 Time = 0.427 Interaction = 0.356	0.138 0.718 0.785
LIFT	21.16 ± 4.93	23.75 ± 5.44	0.50 (0.34 to 0.66)		
Control	23.36 ± 5.44	22.66 ± 6.02	-0.12 (-0.28 to 0.03)		
***5TSTS (s)***					
HIFT	15.11 ± 5.33	12.74 ± 4.78	-0.47 (-0.62 to -0.31)	Group = 0.891 Time = 0.331 Interaction = 0.455	0.017 1.216 0.583
LIFT	14.85 ± 3.24	13.08 ± 4.11	-0.48 (-0.63 to -0.32)		
Control	14.54 ± 3.88	14.79 ± 4.45	0.06 (-0.09 to 0.21)		

HIFT: high-intensity functional training; LIFT: low-intensity functional training; MMSE: Mini–mental state examination; SDMT: symbol-digits modalities test; CVLT-II: California Verbal Learning Test-II; BVMT-R: Brief Visuospatial Memory Test–Revised; 10MWT: 10 m walking test; 6MWT: six minutes walking test; SSST: six spot step test; TUG: timed up and go; 5TSTS: five-time sit to stand test; †: indicated significant within-group pre-post change; #: indicated significant main or interaction effect; *: indicated significant improve compared to control; Effect size (*d*) (< 0.02, 0.02 > < 0.49, 0.05 > < 0.79 and > 0.8, respectively indicated trivial, small, moderate and large effect size)

### Biochemical and physiological outcomes

There was a significant time main effect (*p* = 0.003), group main effect (*p* = 0.001), or group by time interaction effect (*p* > 0.001) for FBG. When examining within-group pre-post changes, we observed a significant improvement in FBG for the HIFT (*ES=* -1.33, *95% CI*, -1.50, -1.16, *P* > 0.001) and LIFT groups (*ES=* -0.96, *95% CI*, -1.12, -0.80, *P* > 0.001) but not for the control group (*ES* = 0.14, *95% CI*, -0.01, 0.30, *P* = 0.446). Effect size estimates for FBG demonstrated that the overall effect size and the lower and upper confidence interval bounds for the HIFT and LIFT groups were “*large*,” while the nonsignificant changes for the control group were deemed “*trivial*.” Between-group comparisons demonstrated that FBG changes in the HIFT and LIFT groups were significant vs. the control group (*both*, *P* > 0.001).

Insulin showed a significant time main effect (*p* = 0.007), group main effect (*p* = 0.021), or group by time interaction effect (*p* = 0.003). Within the group, pre-post changes demonstrated a significant improvement in insulin for the HIFT (*ES=* -0.89, *95% CI*, -1.05, -0.83, *P* > 0.001) and LIFT groups (*ES=* -0.76, *95% CI*, -0.92, -0.60, *P* = 0.002) but not for the control group (*ES* = 0.14, *95% CI*, -0.01, 0.30, *P* = 0.446). Effect size estimates for insulin demonstrated that the overall effect size and the lower and upper confidence interval bounds for the HIFT group were “*large*,” and those for the LIFT group were “*moderate*,” while the nonsignificant changes for the control group were deemed “*trivial*.” Between-group comparisons demonstrated that insulin changes in the HIFT (*P* > 0.001) and LIFT (*P* = 0.008) groups were significant vs. the control.

A significant time main effect (*p* = 0.001), group main effect (*p* = 0.001), or group by time interaction effect (*p* = 0.001) was observed for HOMA-IR. When examining within-group pre-post changes, we observed a significant improvement in HOMA-IR for the HIFT (*ES=* -1.61, *95% CI*, -1.78, -1.43, *P* > 0.001) and LIFT groups (*ES=* -1.49, *95% CI*, -1.66, -1.32, *P* > 0.001) but not for the control group (*ES* = 0.11, *95% CI*, -0.04, 0.26, *P* = 0.519). Effect size estimates for HOMA-IR demonstrated that the overall effect size and the lower and upper confidence interval bounds for the HIFT and LIFT groups were “*large*,” while the nonsignificant changes for the control group were deemed “*trivial*.” Between-group comparisons demonstrated that HOMA-IR changes in the HIFT and LIFT groups were significant vs. the control group (*both*, *P* > 0.001).

HbA1c demonstrated a significant time main effect (*p* = 0.013) and group by time interaction effect (*p* = 0.021) but not a group main effect (*p* = 0.021). Within-group pre-post changes showed a significant improvement in HbA1c for the HIFT (*ES=* -1.01, *95% CI*, -1.18, -0.85, *P* > 0.001) and LIFT groups (*ES=* -0.83, *95% CI*, -0.99, -0.67, *P* > 0.001) but not for the control group (*ES* = 0.11, *95% CI*, -0.04, 0.26, *P* = 0.519). Effect size estimates for HbA1c demonstrated that the overall effect size and the lower and upper confidence interval bounds for the HIFT and LIFT groups were “*large*,” while the nonsignificant changes for the control group were deemed “*trivial*.” Between-group comparisons demonstrated that HbA1c changes in the HIFT (*P* > 0.001) and LIFT (*P* = 0.005) groups were significant vs. the control.

SBP only demonstrated a significant time main effect (*p* = 0.041) but not a group main effect (*p* = 0.177) or group by time interaction effect (*p* = 0.091). On the other hand, within-group pre-post changes showed a significant improvement in SBP for the HIFT (*ES=* -0.82, *95% CI*, -0.98, -0.58, *P* > 0.001) and LIFT groups (*ES=* -0.58, *95% CI*, -0.74, -0.42, *P* = 0.023) but not for the control group (*ES* = 0.15, *95% CI*, 0.03, 0.26, *P* = 0.492). Effect size estimates for SBP demonstrated that the overall effect size and the lower and upper confidence interval bounds for the HIFT group were “*large*,” and those for the LIFT group were “*moderate*,” while the nonsignificant changes for the control group were deemed “*trivial*.”

**Fig. 2:**
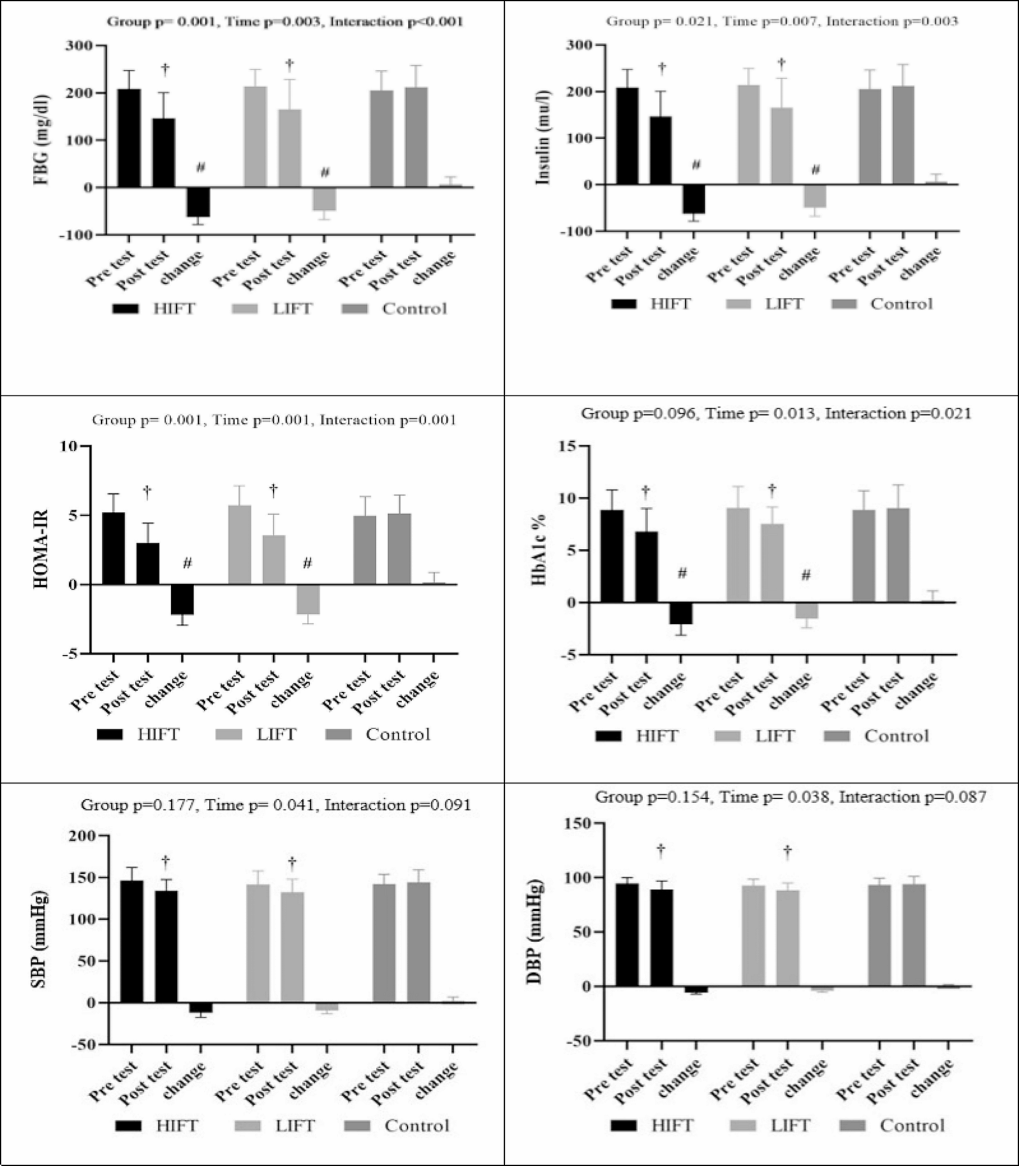
Pre-post changes in biochemical and physiological outcomes in the study groups. HIFT: high-intensity functional training; HbA1c: hemoglobin A1c; LIFT: low-intensity functional training; SBP: systolic blood pressure; HOMA-IR: homeostasis model assessment of insulin resistance; FBG: fasting blood glucose; DBP: diastolic blood pressure; †: indicated significant within-group pre-post change; #: indicated significant improvement compared to control; Effect size (*d*) (< 0.02, 0.02 > < 0.49, 0.05 > < 0.79 and > 0.8, indicated trivial, small, moderate and large ES respectively)

Similarly, DBP only demonstrated a significant time main effect (*p* = 0.038) but not a group main effect (*p* = 0.154) or group by time interaction effect (*p* = 0.087). On the other hand, within-group pre-post changes showed significant improvement in DBP for the HIFT (*ES=* -0.83, *95% CI*, -0.99, -0.65, *P* > 0.001) and LIFT groups (*ES=* -0.65, *95% CI*, -0.81, -0.49, *P* = 0.014) but not the control group (*ES* = 0.10, *95% CI*, -0.06, 0.25, *P* = 0.606). Effect size estimates for DBP demonstrated that the overall effect size and the lower and upper confidence interval bounds for the HIFT group were “*large*,” and those for the LIFT group were “*moderate*,” while the nonsignificant changes for the control group were deemed “*trivial*.” (Fig. 2).

## Discussion

This pilot randomised trial examined the feasibility, safety, and preliminary efficacy of HIFT compared to LIFT for elderly T2D patients with cognitive impairment in cognition (processing speed, memory, learning, and attention), fitness (balance, walking endurance, gait speed, strength, and mobility) and biochemical outcomes (FBG, insulin, HOMA-IR, HbA1c, SBP, and DBP). We proposed a high-intensity low-volume functional training program including physically, biochemically and cognitively challenging exercises to enhance disease profiles and every day functional and cognitive capabilities in elderly with T2D [47–49]. We hypothesised that a group of elderly T2D patients suffering cognitive impairment can use HIFT advantages more than those of LIFT. The present work is the first study, as far as we know, that is a comprehensive examination of the efficacy and feasibility of HIFT vs. LIFT for elderly T2D-related physical disability, biochemical disorders, and cognitive impairment. The results in terms of feasibility was a VAS to measure a positive global rating on the interventions, evaluating the duration as enough, finding the goals achievable, and feedback given in the session, and finding the training enjoyable. The plan to conduct a complete study based on the pilot study can be made based on the feasibility studies rather than the secondary outcomes [50]. However, the intervention hare not examined in terms of feasibility in elderly patients with T2D. These exercises have been found efficient way to enhance physical functioning in elderly with T2D. Both HIFT and LIFT modalities are inexpensive with easy high-application exercise methods. The benefits of both HIFT and LIFT modalities in terms of physical functionality with high application, like those reported by the public, were motivations for choosing the modality programs. Still, the interventions have not been examined so far in terms of feasibility in elderly patients with T2D. Six weeks of both HIFT and LIFT modalities was practical and without risk for a small group of elderly patients with T2D and cognitive impairment. Here, it was found that the adherence rate was high toward the training protocol of 91% (262 of 288 sessions) for the HIFT group and 87.5% (420 of 480 sessions) for the LIFT group. This shows the high safety, feasibility, and good experiences of both exercise training protocols, and feasibility is also supported by the low dropouts. The findings are also consistent with other studies illustrating functional exercise training in elderly subjects [51, 52] as feasible with a high rate of adherence. Additionally, some pilot feasibility studies have mentioned a low rate of adherence in TD2 elderly subjects [53, 54]. These inconsistent findings can be attributed to many factors. First, the training sessions were held at the place of the Diabetes Association, which had suitable conditions in terms of ease of access, training time, and reception of patients. Second, it seems that the frequency and term of both exercise training programs were good; that is, lack of motivation, intensity/load of training, the volume of training, and low adherence rates of the exercises [55] might explain the high rate of adherence to exercise training. Moreover, making a comparison between different studies is not easy given the different methods for defining and measuring adherence rates using by different studies [54].

The cognitive outcomes showed significant improvement in MMSE and Stroop test scores in both the HIFT and LIFT groups. The changes in the Stroop test score in both groups were also significant compared to the control group. However, we found significant improvement in SDMT, CVLT-II, and BVMT-R scores only in the HIFT group. Among physical fitness outcomes, the 6MWT significantly improved in both the HIFT and LIFT groups. The changes in the 6MWT in both groups were also significant in comparison with the control group. Moreover, balance, 10MWT, SSST, TUG, and hand grip significantly improved only in the HIFT group. Additionally, FBG, insulin, HOMA-IR, HbA1c, SBP, and DBP significantly improved in both the HIFT and LIFT groups. The changes in FBG, insulin, HOMA-IR, and HbA1c in both groups were also significant compared to the control group.

Tt is well known that different types of exercise training have diverse structural and functional effects on the brain. It showed that 8 weeks of combined aerobic/resistance training enhanced specific domains of cognitive functions of elderly with T2D, like inhibitory control, cognitive flexibility, attention/concentration, and working memory [15]. Similar improvements in global cognition, learning, memory, processing speed, and attention were reported after aerobic [56, 57] and resistance training [58, 59]. However, it seems that functional training can improve the cognitive function of elderly T2D patients due to the creation of more mental challenges. Functional training is a special intervention for rehabilitation that is used in more practical scenarios to improve the performance of daily activities and includes aerobic exercises, resistance exercises, balance exercises, proprioceptive integration exercises, body positioning exercises, and core muscle stability exercises [23, 24]. A growing body of evidence shows how functional exercises create adaptation in the structure and function of the brain [25, 26]. A systematic review reported that HIFT can improve global and subdomain cognitive function in elderly suffering cognitive impairment [60]. It is recommended that for elderly with T2D, exercise intensity is one of the most important training variables that must be carefully controlled. Studies have shown that high-intensity exercises have more effects on the cognitive performance of elderly with T2D [15, 16]. Regarding the intensity of training, the key role of lactate should be mentioned. Lactate is a main regulator of metabolism, and Brooks (2020) proposed lactate as a fulcrum of metabolic regulation in the body [61]. The evidence presented in a review study by Coco et al. (2020) shows that lactate in the brain acts not only as an energy substrate but also as an angiogenic agent, neuromodulator, and protection against stress [62]. Muller et al. (2020) also showed in a review study that the increase in lactate caused by intense exercise causes a significant enhancement of brain-derived neurotrophic growth factor (BDNF) and has a major role in neuroplasticity and the protection of the nervous system [63]. Therefore, considering the lactate threshold as an important indicator in adjusting the intensity of exercise training to achieve better results should be considered [20].

Additionally, as the findings showed, exercise training has great potential to improve diabetes-related biochemical indices such as FBG, insulin, HOMA-IR, HbA1c, and blood pressure. Based on the importance of exercise intensity, Gordon et al. (2009) [18] and Irwin et al. (2009) [64], in meta-analysis reviews, suggested that higher exercise intensity can be an important factor for blood glucose control. Importantly, improvement in the metabolic profile of elderly T2D patients is related to their cognitive functions. It was reported that changes in IR were related to memory amelioration in older T2D patients; stilly, the actual mechanism is not known yet [15]. Many hypotheses are assumable. For instance higher levels of plasma insulin decreases transport of insulin between blood-brain barrier, which can be due to dysfunctional transport mechanism in endothelial cells. Thus, lower insulin signalling can attributed to a lower cholinergic activity and impaired potentiation (long-term) of the hippocampus, which are important for formation of memories [65]. Enhancement of FBS and variations in the processing speed were correlated significantly and variations in HbA1c was correlated to changes in the processing speed, which can indicate that amelioration of high glucose levels in the blood is related to the enhancement in executive function. Additionally, FBG and blood pressure were significantly correlated with visual search, mental flexibility, speed of processing, scanning, and executive function as sensitive indicators of cognitive impairment in the elderly [66]. Studies have indicated a relationship between diabetes and slower speeded tasks mostly those that measure reaction time or perceptual speed [67].

Consistent with our findings, studies showed that different exercise training modalities can improve physical function in elderly T2D patients [68–70]. As a key point in the meta-analysis article by Patterson et al. (2010), they indicated that high-intensity exercise training significantly improves the physical performance of the elderly compared to moderate-intensity training [19]. Sanders et al. (2019), in a meta-analysis study of systematic review nature, reported that for elderly people with cognitive impairment, an exercise program with shorter sessions and higher intensity results in a significant improvement in physical performance [20]. Another key indicator in improving cognitive function is improving physical function. Apparently, there is a relationship between fitness enhancement and changes in cognitive function with physical training [68]. Similar to the significant improvement in the 6MWT in the present study, after exercise training, a higher enhancement in aerobic fitness (VO_2peak_) were significantly associated with greater changes in cognition. In addition, it seems that a higher fitness level was related to lower loss of cognitive function in the elderly [71].

In general, given that functional exercise is a more challenging exercise and based on similarities with the activities of daily living, a variety of training and multijoint movements that increase neuromuscular coordination have a great effect on cognition [23, 24]. It seems that functional exercise, through increasing neuroplasticity, functional connectivity, microstructural change, homologous region adaptation, and extra region recruitment, can improve cognitive function [72]. Interestingly, high-intensity training, such as HIFT, has greater metabolic benefits, such as improvement in FBG and IR, blood pressure control, better vascular function, and a decrease in inflammation, which are significantly related to cognitive improvements [65]. Additionally, high-intensity training, such as HIFT through lactate mediation, can affect brain neurotrophic and growth factors and, in this way, improve cognitive performance [61]. Improving physical fitness following high-intensity training, such as HIFT, through the positive psychological effects of reducing fatigue, lowering the risk of falling, increasing the quality of life, and lowering the need for hospitalisation can improve cognitive performance [68].

This study has limitations including small sample size. Also, the authors had limitations for the evaluation of proteins and indicators related to cognitive function. Patients’ use of multiple and diverse drugs is another limitation beyond the control of the authors.

## Conclusions

This pilot study showed that high-intensity low-volume vs. low-intensity high-volume functional training is a safe, feasible, and effective way to enhance aspects of physical, biochemical, and cognitive function in older T2D patients who have cognitive impairment. HIFT can improve variables related to cognitive function in elderly T2D patients, including global cognition, attention, speed, memory, and learning, directly or indirectly by improving metabolic and functional status. In addition, this pilot study gives primary proof-of-concept data for a properly powered randomised controlled trial (RCT) of HIFT vs. LIFT in a bigger sample size of elderly T2D patients with cognitive impairment.

## Data Availability

The datasets used and/or analysed during the current study are available from the corresponding author on reasonable request.

## Abbreviations

T2D: Type-2 diabetes
HIFT: High-intensity functional training
LIFT: Low-intensity functional training
RCT: Randomised controlled trial
T3D: Type-3 diabetes
MCS: Metabolic cognitive syndrome
HbA1c: Hemoglobin A1c
FBG: Fasting blood glucose
MMSE: Mini–Mental State Examination
BMI: Body mass index
SDMT: Symbol Digit Modalities Test
CVLT-II: California Verbal Learning Test Second Edition
BVMT-R: Brief Visuospatial Memory Test-Revised
SCWT: Stroop Color and Word Test
6MWT: Six-minute walking test
SSST: Six spot step test
TUG: Timed up-and-go test
5TSTS: Five-time sit-to-stand test
HOMA-IR: Homeostasis model assessment of insulin resistance (HOMA-IR)
SBP: Systolic blood pressure
DBP: Diastolic blood pressure
HRR: Heart rate reserve (HRR)
ANOVA: Analysis of variance
